# Frailty and Sarcopenia in Older Patients Receiving Kidney Transplantation

**DOI:** 10.3389/fnut.2019.00169

**Published:** 2019-11-12

**Authors:** Ilaria Gandolfini, Giuseppe Regolisti, Alberto Bazzocchi, Umberto Maggiore, Alessandra Palmisano, Giovanni Piotti, Enrico Fiaccadori, Alice Sabatino

**Affiliations:** ^1^UO Nefrologia, Azienda Ospedaliero-Universitaria di Parma & Dipartimento di Medicina e Chirurgia, Università di Parma, Parma, Italy; ^2^Dipartimento di Medicina e Chirurgia, Università di Parma, Parma, Italy; ^3^Diagnostic and Interventional Radiology, IRCCS Istituto Ortopedico Rizzoli, Bologna, Italy

**Keywords:** frailty, sarcopenia, kidney transplant, elderly, disability, malnutrition

## Abstract

Kidney transplantation is the treatment of choice for most of the patients with end-stage renal disease (ESRD). It improves quality of life, life expectancy, and has a lower financial burden to the healthcare system in comparison to dialysis. Every year more and more older patients are included in the kidney transplant waitlist. Within this patient population, transplanted subjects have better survival and quality of life as compared to those on dialysis. It is therefore crucial to select older patients who may benefit from renal transplantation, as well as those particularly at risk for post-transplant complications. Sarcopenia and frailty are frequently neglected in the evaluation of kidney transplant candidates. Both conditions are interrelated complex geriatric syndromes that are linked to disability, aging, comorbidities, increased mortality, and graft failure post-transplantation. Chronic kidney disease (CKD) and more importantly ESRD are characterized by multiple metabolic complications that contribute for the development of sarcopenia and frailty. In particular, anorexia, metabolic acidosis and chronic low-grade inflammation are the main contributors to the development of sarcopenia, a key component in frail transplant candidates and recipients. Both frailty and sarcopenia are considered to be reversible. Frail patients respond well to multiprofessional interventions that focus on the patients' positive frailty criteria, while physical rehabilitation and oral supplementation may improve sarcopenia. Prospective studies are still needed to evaluate the utility of formally measuring frailty and sarcopenia in the older candidates to renal transplantation as part of the transplant evaluation process.

## Introduction

Kidney transplantation is the treatment of choice for most of the patients with end-stage renal disease (ESRD). In fact, it improves quality of life (1, 2), life expectancy (3–5), and has a lower financial burden to the healthcare system in comparison to dialysis (6). The increased prevalence of ESRD in parallel with stable or decreased organ availability has led to an increase in the average waiting time for transplantation. In addition, every year more and more older patients are candidates for the enrollment in the kidney transplant list (7). Although in this case patient and graft survival are lower than those for younger recipients (7), kidney transplant guarantees, also in this category, a survival advantage (4, 8) and better quality of life (9, 10) as compared to dialysis. Therefore, it is crucial to select which older patient may benefit from renal transplantation, and which are at high risk for post-transplant complications.

The suitability for transplantation is based on the evaluation of the balance between the risks faced by the patient undergoing the procedure and the risks associated with staying on dialysis. Sarcopenia and frailty are frequently neglected in the evaluation of kidney transplant candidates. However, these two geriatric syndromes have many overlapping causes and consequences with major impact in this specific clinical setting.

As a matter of fact, sarcopenia, which is characterized by a loss of muscle mass and function, correlates with increased mortality and graft failure (11, 12) while frailty, a measure of reduced functional reserve and increased vulnerability of the organism to stressful events, is associated with an increased incidence of delayed graft function (13), reduced tolerance to immunosuppressive drugs such as mycophenolate mofetil (14), increased risk of early rehospitalization (15) and mortality in patients with kidney transplantation (16–18). In the present review, we will thoroughly discuss the pathogenesis and epidemiology of frailty and sarcopenia in older transplant candidates and recipients.

## Frailty Definition and Pathogenesis

Even though the concept of frailty derives from the necessity born in the geriatric field of assessing “biological age” and predicting outcomes in older people (14), now it is spreading across many different clinical contexts. Older people are at increased risk for mortality, morbidities and hospitalization (19). However, the chronological age cannot be considered the only precise and linear measure of the risk of frailty and sarcopenia, and many factors are contributing to increasing the risk of these complications. Moreover, even after taking into consideration the different comorbidities, it is not straightforward to predict the vulnerability to adverse outcomes in the individual elderly subject.

From this gap of knowledge, the concept of frailty was born. The frailty syndrome is characterized by a reduced functional reserve, vulnerability to stressors and increased risk for adverse clinical outcomes (20–24). The main innovation of the frailty concept is the predictive power of developing future complications in response to hypothetic stressors.

It has been suggested that this clinical syndrome is the nonlinear result of multiple dysfunctional biological systems, regardless of the individual dysfunction, chronic disease, and chronological age progressively dysreguling the organism homeostasis (25–33). First of all, chronological age itself has been associated with progressive dysregulation of the organism homeostasis. Mild proinflammatory state (i.e., as revealed by increased IL-6, C-reactive protein [CRP], leukocyte count, and lymphocyte activation pathways), hypercoagulation, anemia, impaired endocrine system, micronutrient deficiencies, neuromuscular deficits have been also associated with the development of frailty (22, 26, 34–37). Moreover, abnormal metabolic systems can interact with the above factors and, increasing the risk for frailty (i.e., abnormal IGF-1, glucose intolerance, DHEA-S and vitamin D) (38–40). Depression and cognitive impairment are additional risk factors for the development of the frailty syndrome (41–44).

**Figure 1:**
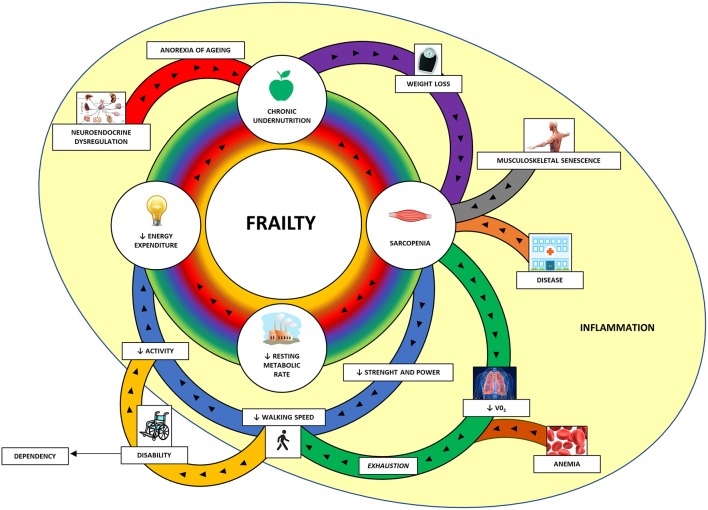
The cycle of frailty (45). The four key factors of frailty (in the central circle) are sarcopenia, lower resting metabolic rate, lower energy expenditure, and chronic undernutrition. These factors are interconnected and amplifying the process and it is hard to distinguish the first factor that started it. In the peripheral area are represented the contributors to these factors. The main contributors to sarcopenia are weight loss (through protein and micronutrient deficit and increased catabolism), musculoskeletal senescence and various diseases. Sarcopenia itself, through the reduction of muscle strength and power, provokes one of the key features of the frailty assessment: the reduced walking speed. The diaphragm and visceral muscle sarcopenia along with anemia are responsible for reduced oxygen uptake (VO_2_) and subsequent exhaustion, which contribute in slowing the walking speed. Reduced walking speed is associated with lower physical activity and subsequent lower energy expenditure. In some cases, the marked reduced walking capacity can configure a disability with loss of independency and need for assistance. Neuroendocrine dysregulation, frequently observed in older people, can reduce the appetite (anorexia of aging) and contribute to chronic undernutrition along with the lower energy expenditure with subsequent weight loss. A mild and chronic inflammation can have a negative impact in these processes. VO_2_, oxygen uptake.

Frailty etiology and progression has a spiraling nature (Figure 1), depending on the total number of the dysfunctional systems involved, rather than on the severity of each system dysfunction (37). Therefore, the frail organism has to find a new precarious balance among these systems that can be disrupted easily by stressors leading to adverse outcomes. The major obstacle to the success of such concept was the absence of a simple, reliable and standardized method aimed at screening for frailty (19, 46–48).

Since frail patients frequently have reduced lean body mass, endurance, balance, strength, and walking speed, as well as low physical activity, all of the frailty scores include measurement of self-reported physical activity and tests for directly quantifying physical activity and sarcopenia (37, 45, 49, 50). In particular, the Fried phenotype score, the most well-known frailty assessment, is based on five components of physical impairment in older adults: weakness (measured by grip strength), slowness (measured by walking speed), low level of physical activity in the past 2 weeks, self-reported exhaustion, and unintentional weight loss (measured by a questionnaire) (37, 51, 52). Individuals are considered frail if 3 or more of the listed components are present, pre-frail if at least one of the components is present or non-frail (zero component) (Table 1). This test is therefore able to identify easily the presence and score the degree of frailty, and to predict negative clinical outcomes, such as the incidence of falls, worsening mobility, hospitalization, and death. The robustness of the Fried score have been documented in the large Cardiovascular Health Study (CHS), and the test has been successfully applied to patient cohorts of Women's Health and Aging Studies (40).

**Table 1:** Measurement and Definition of Frailty Components using the Fried criteria (45).

**Component**	**Positive assessment (=1 point each)**	**Methods**
Shrinking	• Unintentionally loss of 10 pounds or more in the last year	The current weight is asked to the patient. The previous year weight is asked to the patient or derived from medical records.
Exhaustion	• Feeling that “everything I did was an effort” OR “I could not get going” for 3 or more days in the past week	The *CES-D Questionnaire*
Physical activity	• Men who expended <383 Kcals/week • Women who expended <270 Kcals/ week	*The short version of the Minnesota Leisure Time Activity questionnaire* is used to assess frequency of physical activities. Physical activity is converted to Kcals/week expended using a standardized algorithm (number of days physical activity in the past 2 weeks × duration of activity in minutes × Kcals burned per minute).
Walking speed	• Men height ≤ 173 cm that required ≥ 7 s • Men height > 173 cm that required ≥ 6 s • Women height ≤ 159 cm that required ≥ 7 s • Women height > 159 cm that required ≥ 6 s • Patients who are unable to complete the test due to physical limitations	Patients are timed while walking 15 feet. Stratified by gender and height.
Grip strength	• Men BMI ≤ 24 cutoff ≤ 29 • Men BMI 24.1–28 cutoff ≤ 30 • Men BMI>28 cutoff ≤ 32 • Women BMI ≤ 23 cutoff ≤ 17 • Women BMI 23.1–26 cutoff ≤ 17.3 • Women BMI 26.1–29 cutoff ≤ 18 • Women BMI>29 cutoff ≤ 21 • Patients who are unable to complete the test due to physical limitations	Grip strength is measured using a Jamar hand-held dynamometer. The cut-offs (kg) are gender- and BMI-specific.

*Each of the 5 components was scored as 0 or 1 representing the absence or presence of that component. The aggregate frailty score was calculated as the sum of the component scores (range 0–5). CED-D Center for Epidemiologic Studies Depression Scale; BMI, body mass index*.

In consideration of the relationship between chronic disease and frailty, some authors took into consideration the presence and severity of chronic diseases in their “Frailty Index” (19, 53–57). This score appears more sensitive and precise to estimate biological age, however its clinical application has been hampered by time consumption and the lack of distinguishing frailty from comorbidity and disability. The relationship between chronic disease and frailty is not clear and although they often share the same etiology and biological pathways as well as many chronic conditions contribute to frailty, the presence of chronic disease does not necessarily mean frailty.

Another critical component of frailty is the cognitive impairment, and for this reason, when the Fried phenotype score was combined with the assessment of cognitive impairment and depression symptoms, a significant association with disability and worsening quality of life at 12 months was observed (41–44). Along with the development of the frailty concept, many authors tried to combine more and more elements in order to increase the predictive power of the score, including memory tests (58), comorbidities and malignancies (59), anthropometric measures, laboratory markers and caregiver reports (60). As a consequence, in face of a minimal increased predictive power, these scores are applicable only by trained geriatric teams, being time consuming in the clinical setting (61).

## Frailty in Transplant Candidates

Thanks to the increased life expectancy and the improvement in medical and surgical management, an increasing number of patients with CKD (62) over 65 years are evaluated for kidney transplant waiting list (63, 64). A number of studies have, indeed, shown a significant improvement in the overall life expectancy (mortality risk 40–60% lower) for older patients receiving a kidney transplant compared to similar waiting-listed patients remaining on dialysis (65–70), even taking into consideration the significantly higher incidence of early mortality (65, 68, 69, 71) and the use of extreme aged (>75 years) cadaveric donors (70).

It is therefore crucial to evaluate at the time of wait listing evaluation which older kidney transplant candidate will benefit from kidney transplantation, and which are subject to an unacceptable risk of adverse events following transplantation (64).

Frailty as defined by Fried has been proposed as a practical and useful assessment in clinical practice evaluation for kidney transplant waiting list (17). The prevalence of frailty is higher among dialysis patients, and it increases with age: 44% of dialysis patients under 40 years are frail, while this prevalence reach 78% among dialysis patients above 70 years. In addition, functional decline is highly prevalent in older patients during the first 6 months of dialysis, especially in the presence of frailty (72). Patients with CKD (62) typically present many risk factors for frailty (71), such as anemia, osteoporosis, cardiovascular disease and chronic inflammation (72, 73). They also experience neuroendocrine changes, like 25-OH vitamin D deficiency, insulin-resistance (73) and low testosterone levels (74), commonly associated with frailty and sarcopenia (32). Since dietary limitations are also part of the medical prescription to CKD patients, in order to reduce the daily intake of phosphorus, potassium, salt and water, this very fact can exacerbate malnutrition and contribute to sarcopenia (75). Moreover, cognitive impairment is common among ESRD patients, with a prevalence of cognitive decline in older patients on hemodialysis between 16 and 38% (76–79).

The investigation of frailty and its effect on transplant outcomes, could help the clinicians to inform the ESRD patients on treatment options and tailor the pre- and post-transplant follow up strategies (80). In fact, a prospective study of ESRD patients on hemodialysis, has linked frailty to a 2.6-fold increase of mortality risk (95% confidence interval, 1.04–6.49; *P* = 0.041). Of note, this important risk increase has been independent of age, sex, comorbidities, and disability (16). Also, in kidney transplant candidates, who are generally selected among “healthier” patients on dialysis, frailty has been significantly associated with mortality on the transplant waiting list (hazard ratio [HR]: 6.7, confidence interval [CI]: 1.5–30.1; *P* = 0.01) independently of age, diabetes, or duration of dialysis (81).

Frail kidney transplant candidates are also less likely to be listed for kidney transplantation compared to non-frail patients (hazard ratio, 0.62; 95% confidence interval, 0.56–0.69; *P* < 0.001), independently of age and other demographic factors (82), and they are by one third less frequently transplanted than their non-frail counterpart (incidence rate ratio, 0.68; 95% confidence interval, 0.58–0.81; *P* < 0.001) (82).

Recently it has been shown that almost 50% of candidates experience a frailty phenotype worsening while on waiting list, and that these changes are associated with increased risk of mortality and longer hospitalization. Monitoring these changes can in fact improve the post-transplant risk stratification, and modify the suitability to kidney transplantation (83).

## Frailty in Transplant Recipients

Patients that are considered frail at the time of transplant have more than 2-fold adjusted risk of mortality (95% CI: 1.01–4.65, *p* = 0.047) (17) compared to non-frail patients, independently of recipient age (13). Frailty has been also associated with a 1.94-fold increased risk for delayed graft function within the first post-transplant week (95% CI, 1.13–3.36; *P* = 0.02) (15), and increased rate of both morbidity and hospital readmission (adjusted RR = 1.61, 95% CI: 1.18-2.19, *p* = 0.002) in the first month after surgery (15). Frail kidney transplant recipients are more susceptible to drug-related adverse events, like in the case of mycophenolate mofetil use, with a 1.29-times (95%CI:1.01–1.66; *P* = 0.04) more frequent need for dose reduction (14), being this latter effect independently associated with a substantially increased risk of death-censored graft loss (aHR, 5.24; 95% CI, 1.97–13.98, *P* = 0.001) (14). Finally, frailty has been recently associated with medium-term cognitive decline (measured by Modified Mini-Mental State Examination) post-kidney transplantation (84).

Despite the increasing evidence of the role of frailty in predicting the pre- and post- transplant outcomes, no current guidelines are indicating a threshold of frailty score at which a patient should be excluded from the waiting list (64).

Notably, after an initial worsening early after kidney transplantation, adult recipients of all ages experience an improvement of the frailty score at 3 months (85). And despite the fact that patients that are considered frail at the moment of transplantation, still present a higher frailty score over time, these patients are more likely to show an improvement after transplantation, showing the reversible state of frailty and the advantage of transplanting this population (85). Moreover, frail patients reported a better improvement in post-transplant health-related quality of life compared to non-frail transplant recipients (frail, 1.35 points/month; 95% confidence interval [CI], 0.65–2.05; non-frail, 0.34 points/month; 95% CI, −0.17–0.85; *P* = 0.02) (86).

Recent KDIGO guidelines suggest that patients should be evaluated for frailty at the time of listing and while on the waiting list, in order to better define inherent risks and enable optimization strategies (87). Further studies are needed to investigate the role of frailty in predicting specific post-transplant outcomes (88) and define the phenotypes of frail transplant candidates who are expected to benefit the most from transplantation over dialysis, in order to tailor the clinical approach to their unique needs and develop interventions to reverse frailty both pre- and post- transplantation (87).

## Sarcopenia Definition and Pathogenesis

Sarcopenia (from the Greek *sarx* = flesh, *penia* = scarcity) is a complex geriatric syndrome associated with the loss of muscle mass and reduced muscle strength (Table 2) (89). Sarcopenia can be defined as primary or secondary. In the first case, it is a sole consequence of aging, while secondary sarcopenia has a multifactorial etiology, and include as possible causes the decline in physical activity, alterations of the endocrine system, presence of comorbidities, inflammation, insulin resistance and nutritional inadequacy (90). However, in a clinical setting characterized by prevalent older subjects, both age-related and diseases related factors play a role in the development of sarcopenia. Reduced muscle strength leads to a reduction in muscle performance, and is a major cause of disability, mortality and other adverse outcomes (91) (Figure 1).

**Table 2:** Criteria for sarcopenia by the European Working Group for Sarcopenia (EWGSOP2) (88).

	**Muscle strength (hand-grip or chair stand)**	**Muscle mass (skeletal muscle mass index by DEXA or BIA)**	**Physical performance (Gait speed)**
Pre-sarcopenia	↓		
Sarcopenia	↓	↓	
Severe sarcopenia	↓	↓	↓

*Cut-off points: Muscle strength: grip strength: <27 Kg for men and <16 Kg for women; chair stand: > 15 s for five rises*.

*Muscle mass: Appendicular skeletal muscle mass (ASM): <20 Kg for men and <15 Kg/women; ASM index (ASM/hight2): <7 Kg/m^2^ for men and <5.5 Kg/m^2^ for women*.

*uscle function: Gait speed <0.8 m/s*.

The 2010 European Working Group on Sarcopenia in Older People (92) recognized for the first time that muscle strength is also an important component of sarcopenia. The group defined the syndrome as a progressive loss of skeletal muscle mass and strength, increasing the risk for the development of physical disability, poor quality of life and death (92–98).

Despite being primarily considered a natural part of aging, the degree of muscle loss is highly variable and depends on the presence of some risk factors. Muscle homeostasis is maintained thanks to a fine balance between the formation of new myocytes, hypertrophy and protein catabolism. This equilibrium is controlled by the nervous, endocrine and immune systems, and is highly affected by nutrition and physical activity (99).

Lack of exercise is believed to be the most important risk factor for the development of secondary sarcopenia (100). Muscle mass picks at around 30 years of age and starts to decline in a rate of 0.5–1% every year, accelerating after 65 years of age (101, 102). A 30% cumulative loss of muscle mass and a 20% loss in muscle cross-sectional area until reaching 80 years have been described (103), being the reduction in muscle fibers and strength more pronounced in people with a sedentary lifestyle.

Hormonal imbalances, including age-related reduction in growth-hormone (GH), testosterone, thyroid hormones, insulin-resistance, reduced IGF-1, and increased cortisol lead to loss of muscle mass and strength (104). Particularly, the reduction of hormonal anabolic signals and the increase in catabolic signals promoted by glucocorticoids and pro-inflammatory cytokines such as TNF-α and IL-6 both contribute to the loss of muscle mass (104).

Inflammatory cytokines activate muscle ring finger 1 (MURF1), which, like atrogin-1, activates the ubiquitin-proteasome degradation system. In addition, they also cause apoptosis through the activation of NF-kB, and activation of the caspase 8. Increased concentrations of TNF-α and IL-6 have been described in older adults (105).

In parallel to the reduced ability to synthetize muscle fibers, a reduction in energy and protein intake is common in the development of sarcopenia. The lack of protein to sustain muscle mass associates with accumulation of oxidized proteins that are scarcely removed from the muscle via the proteolytic system, leading to an increase of non-contractile dysfunctional protein content in the skeletal muscles. This effect is thought to explain, at least in part, why muscle strength is severely decreased in sarcopenia (106).

## Sarcopenia in Kidney Transplant Candidates

Sarcopenia is a frequent finding in kidney transplant candidates (107), in whom muscle loss occurs at a younger age, and more markedly, in comparison to age-matched controls (108, 109). This phenomenon has been associated to nutritional problems, chronic diseases, sedentary lifestyle and drug-related side effects (110, 111), and its prevalence seems to be related with worsening of renal function (112).

Currently available evidence divides the causes of sarcopenia in kidney transplant candidates into two groups: causes related to the kidney disease itself, and causes related to the chronic low-grade inflammatory process typical of patients on dialysis, but also present in earlier stages of the disease (113, 114). Factors related to kidney disease that contribute to the development of sarcopenia include nutritional deficits and consequent malnutrition, vitamin D deficiency, metabolic acidosis, insulin resistance, low physical activity, hyperparathyroidism, and proteinuria (115).

Inadequate nutrient intake is the most important factor that contributes to the development of sarcopenia in these patients. Progressive loss of appetite begins already in the earlier stages of CKD (116), and it worsens in parallel to the loss of renal function. In this clinical setting anorexia can be viewed as the consequence of the complex negative interactions between metabolic signaling, accumulation of uremic toxins, alterations of factors that regulate appetite (such as gastric mediators, adipokines, and cytokines), and altered hypothalamic signaling (116). In addition, CKD patients on dietary nutritional treatment (the so called conservative treatment) usually undergo prolonged restrictions of protein intake, as well as of phosphorus, potassium and sodium; all of these dietetic measures, though aimed at preventing metabolic complications, can set the stage to the development of malnutrition, especially when energy intake is inadequate (117, 118).

Metabolic acidosis is a very frequent complication of ESRD, and represents a powerful stimulus for protein catabolism (119). In fact, metabolic acidosis activates two systems responsible for intracellular protein degradation, the caspase-3 and the ubiquitin-proteasome systems, and may also reduce protein synthesis and promote insulin and GH resistance, thus leading to negative protein balance (120, 121).

Insulin resistance is one of the most important metabolic challenges in kidney transplant candidates. It has been demonstrated that diabetes is a major risk-factor for sarcopenia in hemodialyzed patients, who suffers from increased protein degradation and loss of lean body mass in comparison to non-diabetic patients (122, 123). However, insulin resistance can be observed also in non-diabetic patients on dialysis, and it is associated with increased protein catabolism also mediated by the ubiquitin-proteasome pathway (124). Insulin resistance is also responsible for a decrease in muscle phosphatidylinositol 3 kinase (PI3K), which may explain the overactivation of the ubiquitin-proteasome pathway. Beside of metabolic acidosis, vitamin D deficiency also contributes to the development of insulin resistance by affecting pancreatic insulin secretion (125, 126). Moreover, muscle is also a target organ for vitamin D, increasing calcium influx from cellular membranes and stimulating muscle synthesis when vitamin D binds to its muscular receptor. Low concentrations of vitamin D are associated with muscle atrophy and sarcopenia (127).

Proteinuria as a consequence of glomerular kidney disease could represent an important additional way of protein loss even in the earlier stages of CKD. Chronic inflammation is a frequent finding in ESRD patients; its consequences include an increase in nutritional needs and the development of anorexia through a lack of balance between orexigenic/anorexigenic mechanisms that control the energetic homeostasis of renal patients (128, 129). Many factors are thought to contribute to the pathogenesis of inflammation in ESRD: worsening of renal function by a reduction of the elimination of pro-inflammatory cytokines and uremic toxins, acute and chronic comorbidities, and factors related to the dialytic treatment itself, such as membrane and dialysis fluid bioincompatibility (130).

In fact, recent data show that the duration of hemodialysis before transplantation is highly correlated with the presence of sarcopenia in kidney transplant candidates (131).

Furthermore, evidence suggest a key role for the gastrointestinal tract as a consequence of intestinal dysbiosis and barrier disruption (132–134). The uremic milieu and reduced intake of fibers that are characteristic of CKD/ESRD patients are responsible for negative effects both on the resident microbial population (i.e., dysbiosis,) and in the structure and function of the gastrointestinal tract, enhancing its permeability (107, 132, 134). This dysbiotic environment of CKD/ESRD is characterized by a switch toward a more proteolytic metabolism profile, leading to an increase in protein fermentation (putrefaction), the generation of increased amounts of potentially toxic compounds (i.e., ammonium, thiols, phenols, indoles) that are absorbed into the bloodstream and accumulate in CKD/ESRD patients (107, 134). In addition, the deranged and more permeable intestinal barrier may facilitate bacterial translocation, which is the passage of bacteria or their structural component lipopolysaccharide (LPS) from the lumen to the blood (107, 134), with a final stimulation effect leading to chronic inflammation. Cytokines like TNFα, IL-6, IL-8, and IFN-γ are, in fact, mentioned among the most frequently observed indicators and activators of muscle proteolysis, while CRP seems to be a useful and inexpensive marker of systemic inflammation, despite being nonspecific (135).

Physical activity and function (defined as the capacity to perform activities of daily living), which are key components in the diagnosis of sarcopenia, are markedly reduced in transplant candidates in comparison to age-matched controls (136, 137). Reasons for reduced physical activity are fatigue on dialysis days, lack of time and motivation, physical problems and pain (138–140). In addition, some physical limitations related to the peritoneal dialysis and the presence of central venous catheter may play a role in limiting the enrollment in some sports. In this clinical setting, physical inactivity represents a modifiable risk factor for the development of sarcopenia, and it may cause further increase in the already high cardiovascular risk of these patients (137, 141). Physical exercise, even in dialyzed patients, is able to reduce depressive symptoms, to improve the quality of life, appetite and energy supply (141).

However, despite the aforementioned higher prevalence of sarcopenia among kidney transplant candidates and its correlation with negative post-transplant outcomes, its evaluation is frequently neglected at the time of waiting list.

## Sarcopenia in Kidney Transplant Recipients

The presence of sarcopenia at the time of kidney transplant has been associated with increased mortality, graft failure, and postoperative complications such as infections (11, 12, 142, 143).

Successful renal transplantation is able to correct or improve many of the conditions related to CKD that promotes muscle wasting and sarcopenia, such as metabolic acidosis and chronic inflammation. However, the use of glucocorticoids as immunosuppressive therapy and the improved, but still suboptimal, renal function can continue to propitiate negative changes in body composition of renal transplant recipients. In general, an increase in body weight is frequently observed early following kidney transplantation (144, 145). However, it seems to be predominantly due to an increase in fat mass instead of muscle mass (146–148). In addition, low physical activity, a common finding in transplant recipients (149, 150), resulting from a combination of low exercise capacity and exercise intolerance/barriers that starts already in the pre-dialysis phases of CKD (140, 151), may significantly contribute to the reduction of muscle mass and function. Skeletal muscle dysfunction, particularly reduced muscle strength, is an important contributor to exercise intolerance in renal transplant candidates and recipients (151, 152). In summary, it seems that changes in body composition of transplant recipients are mainly characterized by an early gain in adipose tissue, while the restoration of muscle mass and function seems to be incomplete. The excess of fat mass and the presence of reduced muscle mass found in transplant recipients characterize a condition called sarcopenic obesity (153). Many studies have shown that when combined, sarcopenia and obesity (as assessed by excess fat mass or central obesity, but not BMI values only) may act in a complementary way increasing the risk of mortality, disability, cardiovascular disease, and metabolic impairment (154–158).

## Sarcopenia and Frailty

Although the pathogenesis of frailty and sarcopenia is not fully understood, these two conditions seem to share risk factors, pathways and often contribute to the same negative outcomes. A growing interest in the frailty and sarcopenia definition and pathogenesis has been observed also in kidney transplant field.

Sarcopenia is one of the most important key components of frailty in kidney transplant candidates, and recognizes as pathogenetic factors aging, physical inactivity, malnutrition, acidosis, metabolic/neuroendocrine dysregulation and mild chronic inflammation (159). Once established, sarcopenia can produce a decrease in resting metabolic rate, contributing to the loss of appetite and malnutrition. The skeletal muscle and the diaphragm can become weaker, leading to increase exhaustion (160), physical inactivity and potential disability in a loop way process (32).

Available evidence regarding the prevalence of frailty components in frail community dwelling adults, revealed that reduced gait speed and weakness were the most common positive Fried criteria (43 and 54% respectively) (41). Increased relative risk for developing weakness and low activity was also reported, in comparison to the risk of developing any other frailty component during 7.5 years of follow up in initially non-frail women (161). This finding suggests that having frailty without sarcopenia is theoretically possible, however it is clinically unlikely.

In the general population sarcopenia has been shown to be twice as common as frailty (162). In kidney transplant candidates, which are selected among the healthier dialyzed patients, the prevalence of these two conditions almost overlaps at around 20% (17), however when the whole dialyzed population is analyzed, the prevalence rises up to 20–44% for sarcopenia and 42% for frailty (163, 164).

Both frailty and sarcopenia are considered reversible conditions. Epidemiological and interventional studies showed that multiprofessional treatments based on individual's frailty positive criteria are successful in improving patients' frailty status (165–167). In addition, other studies specifically have shown that it is possible to improve muscular components of frailty, and consequently sarcopenia (168, 169), confirming that frailty and sarcopenia are linked conditions that correlate to musculoskeletal aging.

## Practical Considerations

**Figure 2:**
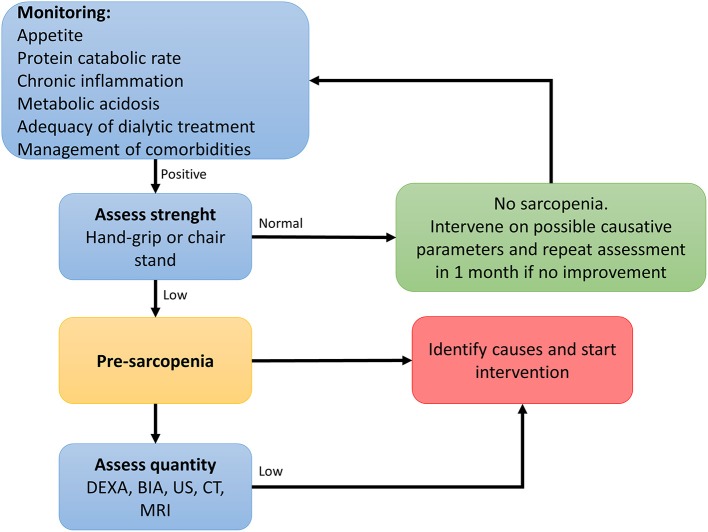
Algorithm to identify cases of sarcopenia in transplant candidates and recipients [adapted from (89)].

In order to prevent or identify subjects at risk for sarcopenia, the EWGSOP2 recommends the use of the SARC-F questionnaire in healthy community living older subjects (89). The SARC-F is a self-reported 5-item questionnaire that reports patients' perceptions and experiences regarding strength, walking ability, chair standing, stair climbing and falls (170). Considering that the SARC-F lacks validation in the renal setting and that sarcopenia in renal patients is mostly secondary, with multiple factors influencing its development, a more comprehensive screening shall be recommended. Periodic appetite assessment tools and food diaries are critical, and allows early intervention when nutrient intake is already slightly reduced (<1 g/Kg/day of protein and <30 Kcal/Kg/day in hemodialysis patients, and <0.8 g/Kg/day in transplant recipients with adequate graft function) (115). In fact, in hemodialysis patients, an intake of protein of <0.8 g/Kg/die and/or calories <25 Kcal/Kg/die in associated with increased risk of malnutrition and sarcopenia (11, 128, 171). Calculation of the protein catabolic rate (PCR) is useful to estimate protein intake in stable patients (172). In transplant recipients not on hemodialysis, PCR can be calculated based on 24 h urinary urea nitrogen excretion, or by urea kinetics in patients on dialysis (172, 173). The recommended energy and protein intake for dialysis patients and transplant recipients are described in Table 3. In addition, considering that patients with ESKD frequently perform blood tests, the combined evaluation of serum albumin and CRP might be useful to assess inflammation. Most importantly, the treatment of conditions related to the renal disease itself that contribute to the increase of catabolism (i.e., proteinuria, metabolic acidosis, hyperparathyroidism, vitamin D deficiency, chronic inflammation) is a key component to prevent the development of sarcopenia in renal patients (115). On this premises, in Figure 2 an algorithm is proposed to identify the presence of sarcopenia among transplant candidates and recipients.

**Table 3:** Energy and protein intake recommendations for patients on dialysis and transplant recipients.

	**Energy**	**Protein**
Dialysis (hemodialysis and CAPD) (172, 174)	30–35 Kcal/Kg/day (adjusting for age and level of physical activity)	≥1.1 g/Kg/day
Transplant recipients with adequate renal function (175)	30–35 Kcal/Kg/da (adjusting for age, gender and level of physical activity)	1.3–1.5 g/Kg/day (first month post-transplant) 0.8–1.0 g/Kg/day (RDI levels as per general population)

## Conclusion

Kidney transplantation is the treatment of choice for most of the patients with ESRD. The suitability for transplantation is based on the evaluation of risks faced by the patient undergoing the procedure and the risks associated with staying on dialysis.

Frailty and sarcopenia are interrelated complex geriatric syndromes that are linked to disability, aging and comorbidities. CKD and ESRD are characterized by multiple factors that set the stage for the development of sarcopenia and frailty. Despite the available evidence showing that frailty and sarcopenia correlate to worse outcomes in transplant recipients, both conditions are frequently neglected in the evaluation of kidney transplant candidates. Prospective studies are needed to evaluate the utility of formally measuring frailty and sarcopenia as part of the transplant evaluation process.

## Author Contributions

IG and AS wrote the first draft of the paper. All authors read and contributed to the final version of the paper.

### Conflict of Interest

The authors declare that the research was conducted in the absence of any commercial or financial relationships that could be construed as a potential conflict of interest.
